# Perioperative fluid balance affects staging of acute kidney injury in postsurgical patients: a retrospective case-control study

**DOI:** 10.1186/2052-0492-2-26

**Published:** 2014-04-03

**Authors:** Yu Horiguchi, Akinori Uchiyama, Naoya Iguchi, Kanaki Sakai, Daisuke Hiramatsu, Kazuyoshi Ueta, Noriyuki Ohta, Yuji Fujino

**Affiliations:** Intensive Care Unit, Osaka University Hospital, 2-15 Yamadaoka, Suita, Osaka, 565-0871 Japan; Department of Anesthesiology and Intensive Care Medicine, Osaka University Graduate School of Medicine, Suita, Osaka, 565-0871 Japan

**Keywords:** Perioperative fluid management, Acute kidney injury, Serum creatinine levels

## Abstract

**Background:**

Although Acute Kidney Injury Network (AKIN) staging is widely used, it has been suggested that classification using serum creatinine levels, which fluctuate because of fluid balance, is not always appropriate for acute kidney injury (AKI) detection. We hypothesized that some patients are misdiagnosed as having no AKI due to dilution resulting from intraoperative infusion, and have worse outcomes than typical patients with no AKI.

**Methods:**

We retrospectively selected patients who did not fulfill the AKI criteria from those who underwent cardiac surgery and remained in an intensive care unit (ICU) for ≥7 days. The patients were divided into two groups: those with AKI (AKI group) and those without AKI (no-AKI group), classified using serum creatinine levels adjusted for fluid balance during the perioperative period. We compared the characteristics and outcomes of the two groups.

**Results:**

After adjustment for serum creatinine, 7 of 26 patients were categorized as having AKI. The AKI group had significantly fewer ventilator-free days during a 28-day period and significantly longer ICU stays than the no-AKI group (5.86 ± 10.0 days vs. 15.6 ± 9.71 days, respectively, *P* = 0.050; 36.4 ± 20.6 days vs. 14.9 ± 10.7 days, respectively, *P* = 0.033).

**Conclusion:**

Adjustment of creatinine level for perioperative fluid balance could improve the accuracy of AKI diagnosis after cardiac surgery.

## Background

Acute kidney injury (AKI) is a common complication in critically ill patients [1] that worsens outcomes [2]. The AKI Network (AKIN) proposed a clinical staging system that utilizes serum creatinine levels [3] and has been shown to predict outcomes [4]. However, a problem with using serum creatinine for AKI staging has recently been identified. Creatinine distributes into the intracellular and extracellular fluid compartments [5], so fluid accumulation may dilute creatinine and delay AKI diagnosis [6]. In patients with acute respiratory distress syndrome (ARDS), AKIN staging after adjustment by fluid balance predicts outcomes more accurately than staging without adjustment, and patients meeting the AKI criteria only after adjustment have a higher mortality rate [7].

In perioperative patients, the incidence of AKI is 10%–30% [1, 8, 9]. Patients also develop positive fluid balance during surgery and on postoperative days (PODs) [10]. AKI staging may be underestimated in these patients, but there have been no reports regarding the AKIN criteria and fluid balance in perioperative patients. In this study, we investigated changes in AKIN staging using serum creatinine levels adjusted by perioperative fluid balance in patients who underwent cardiac surgery, who tend to accumulate more fluid. We used methods reported by Macedo et al. [6] to retrospectively adjust serum creatinine levels based on perioperative fluid balance, and made AKIN stage diagnoses based on adjusted levels in perioperative patients who did not meet the AKIN criteria based on unadjusted levels. We hypothesized that perioperative patients who met the AKIN criteria for stage 1 or higher after fluid adjustment would have poorer outcomes than those who were stage 0 both before and after fluid adjustment.

## Methods

### Study population

We examined patient records to retrospectively investigate patients who underwent cardiac procedures between January 2011 and July 2012, stayed in the intensive care unit (ICU) for at least 7 days, and did not develop AKI as determined by AKIN staging during their ICU stay. Patients with hemodialysis before surgery and patients under 18 years of age were excluded. This research was approved by the Ethics Committee of the Osaka University Graduate School of Medicine (approval number 12191).

### Definition of AKI

We defined AKI using the AKIN consensus definition [3]. AKIN stage 1 disease is an increase in serum creatinine levels of 50% or an absolute increase of ≥0.3 mg/dl over a 48-h window during PODs 1–6. The initial baseline creatinine level was obtained immediately before surgery; subsequently, a new baseline creatinine level, defined as the lowest value during the arbitrary 48-h window, was set before the increase in creatinine levels. We examined the incidence of AKI over the first 7 days of the study. To calculate adjusted creatinine levels, we first estimated the volume of distribution for creatinine levels before surgery, which was equal to total body water (TBW). We assumed that TBW was 60% of the patient's weight before surgery. For each study day, we used the 24-h fluid intake and output to calculate the cumulative fluid balance according to the following equation [6]:

where SCr is the measured serum creatinine level [7].

### Data collection

The patients classified as having no AKI before adjustment were divided into two groups for analysis: no AKI and AKI after adjustment. Proportions were calculated for the frequency of categorical variables in each group. Age, sex, weight, new simplified acute physiology score (SAPSII) [11], type of surgery, time taken for the surgery, serum creatinine levels before surgery and at PODs 1–6, fluid balance and urine output at surgery and on PODs 0–5, ventilator-free days within a 28-day period, ICU stay, hospital stay, serum creatinine levels on the 28th day, and renal replacement therapy on the 28th day were recorded.

### Statistics

Descriptive statistics for categorical variables were expressed as the frequency and percentage, whereas continuous variables were expressed as means ± standard deviations as appropriate. Fisher's exact test was used to compare categorical variables; the *t* test was used to compare continuous variables. Measured unadjusted creatinine levels, adjusted creatinine levels, and fluid balance after surgery were analyzed by a two-way repeated measures analysis of variance. Tukey's *post hoc* test was performed to compare differences between groups on each POD. Tests were two-sided with the alpha level set at 0.05 for statistical significance.

## Results

Twenty-six patients met the inclusion criteria of the study. After adjustment of creatinine levels, 19 patients did not meet the AKI criteria (no-AKI group) and seven patients (26.9%) met the criteria (AKI group). Baseline characteristics of the two groups are shown in Table 1. Preoperative serum creatinine and SAPSII were similar in both groups. Durations of surgery and cardiopulmonary bypass (CPB) were significantly longer in the AKI group than in the no-AKI group (544 ± 164 min vs. 379 ± 134 min, respectively, *P* = 0.041; 316 ± 145 min vs. 129 ± 90.4 min, respectively, *P* = 0.013). There were no significant differences in fluid balance during surgery (6,090 ± 4,790 ml vs. 2,630 ± 2,120 ml, respectively, *P* = 0.11).

**Table 1:** **Patient characteristics**

	AKI group (AKI after adjustment [***n*** = 7])	No-AKI group (no AKI after adjustment [***n*** = 19])	***P*** value
Age, years	57.1 ± 18.7	54.2 ± 20.2	0.73
Male sex	6	13	0.63
Body weight, kg	61.2 ± 5.29	57.4 ± 12.0	0.28
SAPSII	33.9 ± 10.9	30.3 ± 7.17	0.45
Preoperative serum creatinine levels, mg/dl	1.14 ± 1.11	1.00 ± 0.42	0.76
Type of surgery			
VAD implantation	3	8	
CABG	1	6	
Valve operation	2	2	
Heart transplantation	0	2	
TAA replacement	1	0	
Congenital heart disease	0	1	
Duration of surgery, min	544 ± 164	379 ± 134	0.041
Duration of CPB, min	316 ± 145	129 ± 90.4	0.011
Use of CPB	7	12	0.0041
Fluid balance during surgery, ml	6,089 ± 4,789	2,629 ± 2,116	0.11

Data are expressed as means ± standard deviations. *VAD* ventricular assist device, *CABG* coronary artery bypass grafting, *TAA* thoracic aortic aneurysm.

**Figure 1:**
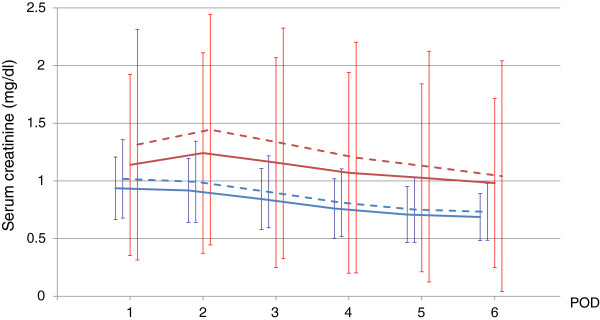
**Unadjusted and adjusted serum creatinine levels.** *Blue solid* and *dashed lines* show unadjusted and adjusted serum creatinine levels in the no-AKI group, respectively. *Red solid* and *dashed lines* show unadjusted and adjusted serum creatinine levels in the AKI group, respectively. There was a significant difference between unadjusted and adjusted serum creatinine levels at PODs 1–6 (*P* < 0.01). There was no significant difference between other groups.

In the AKI group after adjustment, two patients reached AKIN stage 1 at POD 1, and five patients reached stage 1 at POD 2. No patients reached AKIN stage 2 or 3 after adjustment. There was a significant difference in serum creatinine levels and adjusted serum creatinine levels at PODs 1–6 (*P* < 0.01). There were no significant differences among groups (Figure 1).

**Figure 2:**
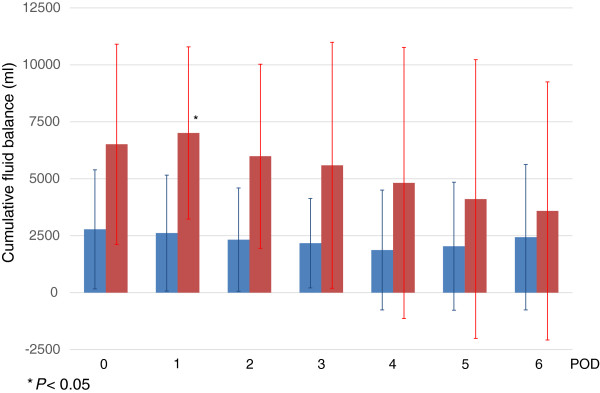
**Cumulative fluid balance from surgery.** *Blue* and *red boxes* show cumulative fluid balance from surgery in the no-AKI and AKI groups, respectively. Cumulative fluid balance from surgery was significantly higher in the AKI group than in the no-AKI group (*P* = 0.0045), especially on POD 1 (7,010 ± 3,780 ml vs. 2,610 ± 2,540 ml, *P* = 0.021).

**Figure 3:**
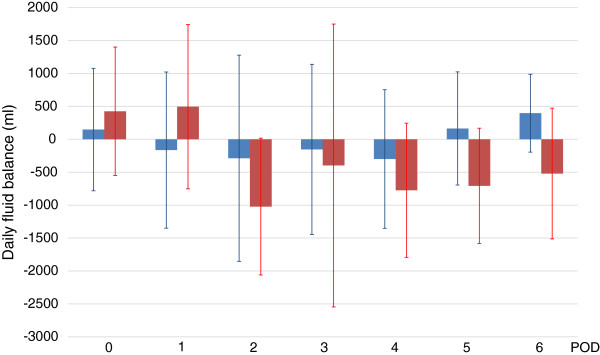
**Daily fluid balance.** *Blue* and *red boxes* show daily fluid balance in the no-AKI and AKI groups, respectively. There was no significant difference in daily fluid balance (*P* = 0.082).

**Figure 4:**
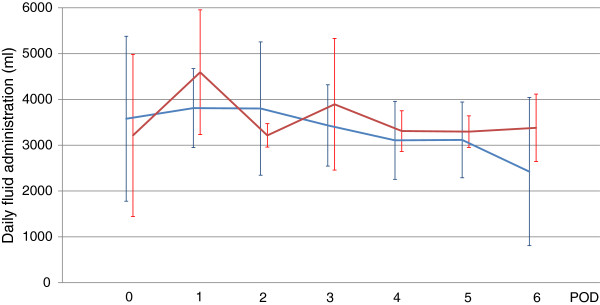
**Daily fluid administration.** *Blue* and *red lines* show total fluid administration in the no-AKI and AKI groups, respectively. There was no significant difference in daily fluid administration (*P* = 0.32).

Cumulative fluid balance from surgery in the AKI group was significantly higher than that in the no-AKI group (*P* = 0.0045), especially on POD 1 (7,010 ± 3,780 ml vs. 2,610 ± 2,540 ml, respectively, *P* = 0.021; Figure 2). There was no significant difference in daily fluid balance (*P* = 0.082; Figure 3) or in daily fluid administration (*P* = 0.32; Figure 4).

**Figure 5:**
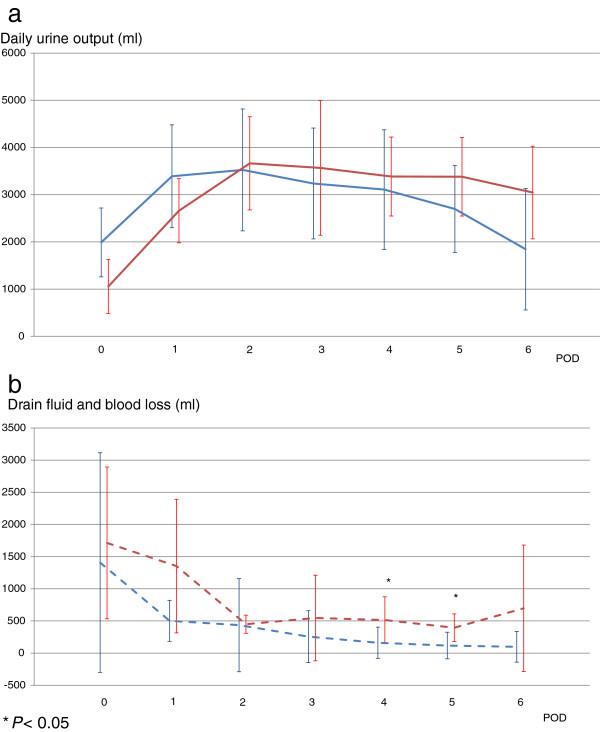
**Daily urine output levels (a) and drain fluid and blood loss levels (b).** *Blue* and *red solid lines* show urine output levels in the no-AKI and AKI groups, respectively. *Blue* and *red dashed lines* show drain fluid and blood loss levels in the no-AKI and AKI groups, respectively. There was no significant difference in urine output (*P* = 0.089). Drain fluid and blood loss levels in the AKI group were significantly higher than those in the no-AKI group (*P* = 0.033). On PODs 4 and 5, these levels were significantly higher in the AKI group (513 ± 362 ml vs. 161 ± 242 ml, respectively, *P* = 0.043; 395 ± 214 ml vs. 117 ± 206 ml, respectively, *P* = 0.014).

Figure 5 shows urine output levels and drain fluid and blood loss levels for the patients during their ICU stay. There was no significant difference in urine output (*P* = 0.089). Drain fluid and blood loss levels in the AKI group were significantly higher than those in the no-AKI group (*P* = 0.033). On PODs 4 and 5, these levels were significantly higher in the AKI group (513 ± 362 ml vs. 161 ± 242 ml, respectively, *P* = 0.043; 395 ± 214 ml vs. 117 ± 206 ml, respectively, *P* = 0.014).

Outcome variables are shown in Table 2. There were significantly fewer ventilator-free days in a 28-day period in the AKI group than in the no-AKI group (5.86 ± 10.0 days vs. 14.9 ± 10.7 days, respectively, *P* = 0.050). ICU stays were significantly longer in the AKI group than in the no-AKI group (36.4 ± 20.6 days vs. 14.8 ± 10.7 days, respectively, *P* = 0.033). There were no significant differences between the AKI and no-AKI groups in hospital stay length (207 ± 176 days vs. 141 ± 225 days, respectively, *P* = 0.44) or serum creatinine levels on the 28th day (1.37 ± 0.93 mg/dl vs. 0.78 ± 0.28 mg/dl, respectively, *P* = 0.15). Two patients in the AKI group needed renal replacement therapy on the 28th day, while no patients in the no-AKI group needed renal replacement therapy; this difference was not statistically significant (*P* = 0.065).

**Table 2:** **Outcome variables**

	AKI group (AKI after adjustment [***n*** = 7])	No-AKI group (no AKI after adjustment [***n*** = 19])	***P*** value
Ventilator-free days within a 28-day period	5.86 ± 10.0	14.9 ± 9.71	0.050
ICU stay, days	36.4 ± 20.6	14.8 ± 10.7	0.032
Hospital stay, days	207 ± 176	141 ± 224	0.45
Serum creatinine levels on the 28th day, mg/dl	1.37 ± 0.93	0.78 ± 0.28	0.14
Renal replacement therapy on the 28th day	2	0	0.065

Data are expressed as means ± standard deviations.

## Discussion

The study indicated that 26.9% of ICU patients diagnosed without AKI after cardiovascular surgery met the AKIN criteria after adjustment of serum creatinine levels based on perioperative fluid balance. These patients underwent more invasive surgery, as reflected by a longer duration of surgery and CPB. Comparison of the AKI group with the no-AKI group after adjustment showed that the AKI group had poorer outcomes, fewer ventilator free-days within a 28-day period, and longer ICU stays than the no-AKI group.

Liu et al. evaluated AKI diagnosis based on serum creatinine levels adjusted for fluid balance in ARDS patients [7]. Of 459 ARDS patients, 131 met the AKIN criteria after adjustment, and the AKI group had a higher mortality rate. The results of the present study showed that the methodology of Liu's study was suitable for situations in which fluid balance changes dynamically, as it does during the perioperative period, in conditions other than ARDS. Although the original AKIN classification defined AKI using relative or absolute changes in serum creatinine levels without accounting for changes in fluid balance levels, both studies suggest that changes in renal function need to be interpreted in the context of fluid management.

Positive cumulative fluid balance is associated with poor outcomes in critically ill patients [12]. In our analysis, the AKI group after adjustment tended to have a more positive fluid balance than the no-AKI group, which may partly explain why the AKI group had poor outcomes. In our study, infusion and transfusion for bleeding, lower urine output due to AKI, or more infusion for treatment and prevention of AKI may have resulted in a more positive balance in the AKI group.

There are some limitations worth noting. First, this study was conducted using a retrospective design in a small number of patients from a single institution, and parameters were not analyzed by subgroups. Therefore, baseline characteristics were similar between the AKI and no-AKI groups, although it is uncertain if other parameters, such as surgery type, bleeding, transfusion, or use of drugs such as catecholamines, may be related to outcomes. Further studies reviewing more patients are needed to confirm and extend our findings. Second, fluid balance during surgery, especially using CPB, could be inaccurate due to incorrect counts of bleeding or infusion. Given that the duration of CPB was longer in the AKI group, fluid balance and adjusted serum creatinine could be less accurate than in the no-AKI group. Effects of adjustment are larger in patients with greater positive fluid balance and higher levels of previous creatinine. In patients with renal dysfunction, creatinine levels before adjustment are likely to be low because fluid balance tends to be more positive during perioperative days. Although there were no significant differences in preoperative creatinine levels between the AKI and no-AKI groups in our study, glomerular filtration rates before surgery might be different. Unfortunately, creatinine clearances were not measured in our patients, and thus, our results might not reflect the precise time courses of renal function deterioration in the perioperative periods. Measurement of creatinine clearance before and after surgery would help to obtain accurate influences of renal dysfunction. Finally, our data were also limited to patients diagnosed as AKIN stage 0 before adjustment for fluid balance. To investigate effects of adjustment in other AKIN stages, further studies are needed to compare all patients, including those diagnosed with AKI before adjustment.

## Conclusion

During perioperative periods, AKIN staging can change after adjustment of creatinine levels for perioperative fluid balance, and adjusted AKIN staging could lead to more accurate AKI diagnosis. Positive fluid balance could underestimate AKI.
